# Impact of Membrane Lipids on UapA and AzgA Transporter Subcellular Localization and Activity in *Aspergillus nidulans*

**DOI:** 10.3390/jof7070514

**Published:** 2021-06-28

**Authors:** Mariangela Dionysopoulou, George Diallinas

**Affiliations:** 1 Department of Biology, National and Kapodistrian University of Athens, 15784 Panepistimioupolis, Greece; mariangelad@biol.uoa.gr; 2 Institute of Molecular Biology and Biotechnology, Foundation for Research and Technology, 70013 Heraklion, Greece

**Keywords:** ergosterol, sphingolipids, phosphoinositide, subcellular trafficking, endocytosis, autophagy

## Abstract

Recent biochemical and biophysical evidence have established that membrane lipids, namely phospholipids, sphingolipids and sterols, are critical for the function of eukaryotic plasma membrane transporters. Here, we study the effect of selected membrane lipid biosynthesis mutations and of the ergosterol-related antifungal itraconazole on the subcellular localization, stability and transport kinetics of two well-studied purine transporters, UapA and AzgA, in *Aspergillus nidulans*. We show that genetic reduction in biosynthesis of ergosterol, sphingolipids or phosphoinositides arrest *A. nidulans* growth after germling formation, but solely blocks in early steps of ergosterol (Erg11) or sphingolipid (BasA) synthesis have a negative effect on plasma membrane (PM) localization and stability of transporters before growth arrest. Surprisingly, the fraction of UapA or AzgA that reaches the PM in lipid biosynthesis mutants is shown to conserve normal apparent transport kinetics. We further show that turnover of UapA, which is the transporter mostly sensitive to membrane lipid content modification, occurs during its trafficking and by enhanced endocytosis, and is partly dependent on autophagy and Hect-type HulA^Rsp5^ ubiquitination. Our results point out that the role of specific membrane lipids on transporter biogenesis and function *in vivo* is complex, combinatorial and transporter-dependent.

## 1. Introduction

The most abundant type of proteins embedded in cell membranes are solute transporters, which are polytypic transmembrane proteins mediating cell nutrition, homeostasis, communication and response to drugs. In eukaryotes, newly made transporters are co-translationally integrated into the lipid bilayer of Endoplasmic Reticulum (ER) [1]. Correctly folded transporters exit the ER packaged in COPII vesicles [2,3] and traffic to their final membrane destination, mostly the Plasma Membrane (PM), via either conventional or unconventional vesicular secretion routes [4,5,6]. In addition to biosynthetic sorting to the PM transporters also undergo regulated endocytic internalization, followed by endosomal recycling to the PM or sorting via the Multivesicular Body(MB)/endosomal compartment to the vacuole (fungi or plants) or lysosome (metazoa) for degradation in response to physiological or stress signals [7]. Consequently, in the course of their biogenesis transporters interact with the different membrane lipids in distinct subcellular compartments (e.g., ER, *cis-*Golgi, *trans*-Golgi, secretory vesicles, sorting or recycling endosomes, vacuole/lysosome). An emerging concept is that membrane lipid composition has high impact on the trafficking, functioning and turnover of transporters [8,9,10,11,12,13]. 

The great majority of membrane lipids can be classified into three major groups, glycerophospholipids, sphingolipids, sterols [14]. Most lipids are synthesized in the ER but their relative abundance changes significantly in different cellular membranes [15]. The interactions of these lipids with transporters or other transmembrane proteins can be of distinct types [16,17,18]. The so-called annular lipid interactions are those involving surrounding lipids contacting the membrane protein as a shell or belt. Structural and functional interactions involve lipids that ‘invade’ clefts between transmembrane helices or cover hydrophobic surfaces, which otherwise would exert deleterious force and local curvatures on the lipid bilayer that cause dysfunction of membrane proteins. Lipids may also have a functional impact on membrane proteins at distance by modifying the fluidity of the membrane [19,20,21] or the fusion and fission of vesicles carrying membrane protein cargoes [22,23]. 

Studies concerning specific roles of distinct lipid species on the functioning of transmembrane proteins remain, extremely challenging [24,25,26]. This is due to technical difficulties in studying the structure and function of membrane proteins in the presence of their native lipid environment and the pleiotropic effects of genetic or pharmacological modification lipid biosynthesis and/or composition *in vivo*. Despite the aforementioned difficulties, there are several cases where specific lipid types have been shown to be critical for transporter biogenesis, function and/or turnover. For example, in *Saccharomyces cerevisiae*, the transport activity and endocytic turnover of the general amino acid transporter Gap1 depends on sphingolipid biosynthesis. More specifically, mutations blocking sphingolipid synthesis lead to reduced accumulation of Gap1 in the PM, loss of transport function and rapid unregulated down-regulation, the later involving ubiquitination of lysine residues normally not accessible to physiologically-elicited turnover [27]. Another example where sphingolipids have been shown to affect targeting and function of a transporter is the case of the major H^+^-ATPase Pma1, where blocks in ongoing sphingolipid synthesis abolish oligomerization of Pma1 in the ER membrane and its association with lipid rafts [28,29,30]. However, non-raft associated Pma1 is exported from the ER but is mislocalized to the vacuole for degradation. Notably, while Pma1 does not need ergosterol to be properly localized in the PM [31], the localization of the yeast tryptophan transporter Tat2 has been shown to be sorted for vacuolar degradation in the ergosterol biosynthesis mutant *erg6* [32]. Under ergosterol depletion, the yeast uracil transporter, Fur4, is also prematurely routed for vacuolar degradation, most probably directly from the Golgi [33]. Both Tat2 and Fur4, similar to some other fungal nutrient transporters, localize in lipid-specific PM microdomains enriched in ergosterol, the so called Membrane Compartment of Can1 (MCC)/eisosomes [21,33,34], suggesting that in such cases deletion of ergosterol might also affect compartmentalization of transporters in specific microdomains. Notice that the sphingolipid-dependent transporters Pma1 and Gap1 strictly avoid MCC/eisosome localization. 

In recent years, direct structural evidence has been accumulated showing that several transporters interact with specific membrane lipids, mostly phospholipids and sterols, and in several cases these interactions seem to regulate transport functioning. For example, in the ABC-type MDR1 or ABCG2 drug efflux transporters, annular lipid interactions involving cholesterol and phospholipids, but also specific structural interactions of the substrate binding site with cholesterol, are crucial for activity. In metazoan neurotransmitter transporters specific for dopamine (DAT), serotonin (SERT) or glutamate (ASCT2), or in the GLUT1 glucose transporter, cholesterol and/or phospholipid binding seems to regulate transporter activities and/or subcellular localization. In the K^+^ inwardly rectifying (Kir) channels interaction with phosphatidylinositol 4,5-bisphosphate (PIP2) triggers a conformational change that tethers the cytoplasmic domain to the transmembrane core and regulates activity. In the mechanosensitive MscL channel interaction with a range of phosphatidylinositol (PI) lipids is critical for protein stabilization, while the ammonia transporter (AmtB) is stabilized by phosphatidylglycerol (PG). These are just some examples on the role of lipids of proteins involved in solute or ion transport. For relative reviews see [12,13] and references therein.

UapA, a H^+^-dependent transporter of xanthine and uric acid in the model fungus *Aspergillus nidulans*, is one of the most deeply studied eukaryotic solute transporter in respect to its regulation of expression, function, substrate specificity, subcellular trafficking, regulated turnover and structure-activity relationships [35]. Its high-resolution crystal structure revealed that UapA is formed from two domains, the core domain and a scaffold domain, and is likely to transport its substrates via the so-called sliding elevator mechanism [36]. The structure also showed that UapA is a homodimer confirming earlier biochemical studies [37]. Analysis of the structure in combination with mutagenesis demonstrated that dimer formation is essential for function [36]. Native Mass Spectrometry (MS) experiments combined with molecular dynamics (MD), mutagenesis and *in vivo* functional analyses recently established that membrane lipids, namely phosphatidylinositol (PI) and phosphatidylethanolamine (PE), interact with specific residues at the dimer interface of UapA and thus play a crucial role in stabilizing the functional UapA dimer [11,38]. Here, we investigate the role of major classes of membrane lipids (ergosterol, sphingolipids or phospholipids) in UapA trafficking, subcellular localization, transport activity and turnover. In parallel, we also study the importance of membrane lipids in a functionally distinct purine transporter, AzgA. AzgA is a purine/H^+^ symporter, that defines a group of transporters present in bacteria, fungi and some plants, structurally similar and possibly distantly related to the NAT family [39]. Our study is carried out principally in novel *A. nidulans* mutants related to ergosterol, sphingolipid or phospholipid biosynthesis, but also makes use of chemical inhibitors of ergosterol biosynthesis. 

## 2. Materials and Methods

### 2.1. Media, Strains and Transformation

Standard complete and minimal media (MM) for *A. nidulans* were used (FGSC, http://www.fgsc.net, accessed date: 20 December 2020). Chemicals were obtained from Sigma-Aldrich (St. Louis, MO, USA) or AppliChem (Darmstadt, Germany). Glucose 0.1–1% (*w*/*v*) or Fructose 0.1% (*w*/*v*) were used as carbon sources. Ammonium tartrate (10 mM), sodium nitrate (10 mM), uric acid (500 μΜ) and hypoxanthine (500 μΜ) were used as nitrogen sources. Thiamine hydrochloride was used at a final concentration of 10 μM as a repressor of the *thiA* promoter [40]. Itraconazole (ITZ) was used at a final concentration of 1 mg/L. Genetic transformation was performed by generating protoplasts from germinating conidiospores of *A. nidulans*, as described previously [41], using TNO2A7 as a recipient strain [42]. Integrations of gene fusions with fluorescent tags, promoter replacement fusions or deletion cassettes were selected using the *A. fumigatus* markers orotidine-5’-phosphate-decarboxylase (*AFpyrG*, Afu2g0836) and GTP-cyclohydrolase II (*AFriboB*, Afu1g13300). Transformants were verified by conventional PCR analysis. Strains used herein are listed in Supplementary Table S1. Standard genetic crossing was employed for the generation of combinations of mutations and/or fluorescent epitope-tagged transporters. *E. coli* DH5α strain was used for molecular cloning. 

### 2.2. Nucleic Acid Manipulations and Plasmid Constructions

DNA fragments used in the various constructs were amplified from the TNO2A7 strain. Plasmid preparation and DNA gel extraction were performed using the Nucleospin Plasmid and the Nucleospin Extract II kits (Macherey-Nagel, Bethlehem, PA, USA). Genomic DNA extraction was performed as described in FGSC (http://www.fgsc.net, accessed date: 15 February 2021). Restriction enzymes, T4-ligases and phosphatases were from Takara Bio (Kusatsu, Shiga, Japan). DNA sequences were determined by Eurofins-Genomics (Vienna, Austria). PCR amplifications were performed using KAPA Taq DNA and Kapa HiFi polymerases (Kapa Biosystems, Wilmington, MA, USA and Roche Diagnostics, Basel, Switzerland, respectively). All gene cassettes were generated by sequential cloning of the relevant fragments in pGEM-T plasmids using restriction enzyme linked oligonucleotides. The resulting plasmids served as templates to PCR-amplify the relevant linear cassettes using high fidelity polymerases. An exception is the Erg11A deletion cassette which was generated by double joint PCR using overlapping and nested primers [43]. Oligonucleotides used herein are listed in Supplementary Table S2.

### 2.3. Protein Extraction and Western Blots 

Total protein extraction was performed using dry mycelia from cultures grown in minimal media supplemented with NaNO_3_ at 25 °C, as previously described [44]. Total proteins (25–50 μg, estimated by Bradford assays) were separated in 8–10 % (*w*/*v*) polyacrylamide gels. Immunodetection was performed on PVDF membranes (GE Healthcare Life Sciences, Amersham, United Kingdom) using primary anti-FLAG M2 (Sigma-Aldrich, St. Louis, MO, USA), anti-GFP (Roche Diagnostics, Basel, Switzerland), anti-actin (C4) (MP Biomedicals, Santa Ana, CA, USA) and an HRP-linked secondary antibody (Cell Signaling Technology Inc, Danvers, MA, USA). Blots were developed using the LumiSensor Chemiluminescent HRP Substrate kit (Genscript, Piscataway, NJ, USA) and SuperRX Fuji medical X-ray films (FujiFILM, Minato City, Tokyo, Japan). 

### 2.4. Kinetic Analysis

Labelled substrates, [2,8-^3^H]-adenine (20 Ci mmol^−1^) and [8-^3^H]-xanthine (22.8 Ci mmol^−1^) were purchased from Moravek Biochemicals (Brea, CA, USA). Kinetic analyses were performed as recently described in detail in [45]. In brief, uptake was assayed in *A. nidulans* germinating conidiospores (3.5–4 h at 37 °C, at 130–150 rpm, in liquid MM, pH 6.8). Conidiospores were resuspended at a concentration of 10^7^ conidiospores/100 μL. Initial velocities were measured by incubation with concentrations of 0.1 μΜ of labeled substrate at 37 °C. Apparent *K*_m/i_ and *V* values were obtained using labelled substrates at 0.1 μM in the presence of various concentrations (0.5–1000 μM) of non-labelled substrates. *V* values were determined from the initial uptake rate plotted against substrate concentrations and are expressed at a concentration of 10^7^ conidiospores. Values were analyzed using http://www.graphpad.com/scientific-software/prism/ (accessed on 26 June 2021). Background counts are subtracted from the values obtained in a strain lacking the relevant transporter. All transport assays (three replicates) were carried out in at least two independent experiments. Standard deviation was <20%.

### 2.5. Epifluorescence Microscopy 

Samples of conidiospores, germinating to germlings or young hyphae, were prepared essentially as previously described in [46,47], using glass bottom 35 mm μ-dishes (ibidi) in liquid MM for 20–24 h, at 25 °C. In Figure 2, strains carrying knockout (*Δerg11A/thiA_p_-erg11B,* *Δerg5,* *Δerg4*) genes were incubated overnight in 2 mL of liquid MM 1% glucose pH 5.5, containing the appropriate supplements and nitrate as a nitrogen source. Notice that for the strain carrying the repressible allele *thiA_p_-erg11B* together with *Δerg11A,* we do not need to add thiamine as this mutant exhibits a severe growth defect even in the absence of repression of Erg11B, suggesting that ergosterol levels are highly reduced. In fact, addition of thiamine to this mutant leads to an absolute block in germination. For strains carrying the *thiA_p_-pisA* or *thiA_p_-basA* thiamine was added from the onset of culture incubation (*ab initio*) in order to pre-establish repression of expression of membrane lipid biosynthesis proteins, prior to UapA or AzgA derepression. In all cases, UapA and AzgA transcription was initially repressed for the first 14–16 h of growth through the use of ammonium as N source, and then depressed by shift in MM supplemented with nitrate as N source, for the following 4–8 h. In experiments shown in Figures 3 and 5 repression or derepression of UapA or AzgA was achieved through the use of the regulatable *alcA* promoter (*alcA_p_*), which replaced the relative native promoters of transporters. Transcription from *alcA_p_* is repressed in the presence of 1% glucose, but derepressed in the presence of 0.1% fructose. The original strains carrying the *alcA_p_*-UapA-GFP, *alcA_p_*-UapA-K572R-GFP or *alcA_p_*-AzgA-GFP alleles have been described before [47,48]. Here, these alleles were introduced by strain crossing in different genetic backgrounds. In Figures 3A and 5A, concerning the effect of ITZ on the localization of neosynthesized transporters, the drug was added 5 h prior of UapA or AzgA derepression. In Figures 3B and 5B, concerning the effect of ITZ on PM pre-localized UapA or AzgA, conidiospores germinated in 0.1% fructose MM overnight (14 h) to elicit derepression of UapA or AzgA, then 1% glucose was added in order to repress further synthesis of the transporters, and after 2 h ITZ was also added for the next 5 h of incubation. In Figure 6, conditions are essentially as those described for Figure 2, following the localization of *de novo* made transporters. 

FM4-64 (Thermo Fischer Scientific, Waltham, MA, USA) or CMAC staining was according to [49]. Nuclear staining was carried out, according to the supplier’s instructions (Molecular Probes, Eugene, OR, USA), with Hoechst 33,258 (bis-benzimide) [50]. Images were obtained using an inverted Zeiss Axio Observer Z1 (Zeiss, Oberkochen, Germany) equipped with a Hamamatsu Orca Flash 4.0 LT Plus camera (Hamamatsu Photonics K.K., Hamamatsu, Shizuoka, Japan). Contrast adjustment, area selection and color combining were made using Zen lite 2012 software (Zeiss, Oberkochen, Germany) and were further processed in Adobe Photoshop CS4 Extended version 11.0.2. 

## 3. Results and Discussion

### 3.1. Biosynthesis of Phosphatidylinositol (PI), Sphingolipids or Ergosterol Is Essential for A. nidulans Growth 

In order to investigate the role of PI phospholipids, sphingolipids and ergosterol on transporter function, we constructed *A. nidulans* strains where key genes needed for relevant lipid biosynthesis are genetically knocked-out or tightly repressed via a regulatable promoter. Details on the construction of mutants are found in Materials and methods. The genes knocked-out or modified in order to be conditionally repressed are described below (full genotypes found in Supplementary Table S1). 

For blocking PI or sphingolipid synthesis we used conditions that lead to transcriptional repression of the *pisA* and *basA* genes, respectively. The *pisA* gene (AN0913) was identified, herein, as an orthologue of *S. cerevisiae, S. pombe* or *C. albicans PIS1* gene, which encodes phosphatidylinositol synthase, an enzyme absolutely required for biosynthesis of phosphatidylinositol in yeasts [51]. As *Pis1* is essential for growth in most fungi studied, here we constructed a mutant strain that allows conditional tight repression of *pisA* transcription, via the thiamine-repressible *thiA* promoter (i.e., *thiA_p_-pisA*). The *basA* gene (AN0640), a yeast *SUR2* orthologue, has been previously reported to encode a sphingolipid C4 hydroxylase that is absolutely essential for growth in *A. nidulans* [52,53]. Thus, for the present work, we constructed a mutant that also carries a repressible, via the *thiA* promoter, *basA* allele (i.e., *thiA_p_-basA*).

**Figure 1:**
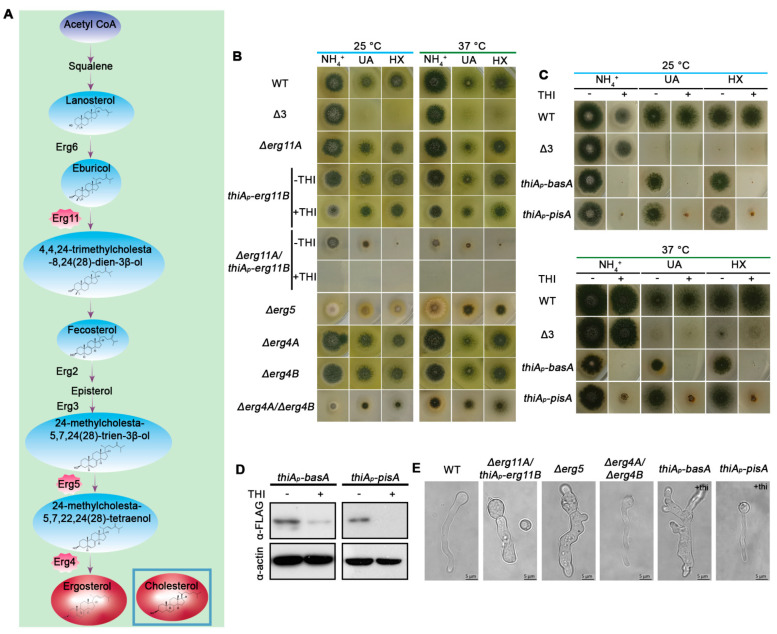
Effect of reduction of biosynthesis of phosphatidylinositol (PI), sphingolipids or ergosterol in *A. nidulans* growth. (**A**) Pathway of ergosterol biosynthesis in Aspergilli (adapted from [55,56]). Only proteins related to the present work are highlighted. Chemical formulas of ergosterol precursors, ergosterol and cholesterol are shown for comparison. (**B**) Growth tests of ergosterol biosynthesis mutants relative to isogenic controls. WT is a standard wild-type *A. nidulans* strain. Δ3 is a stain with total genetic deletions in the three major purine transporters UapA, AzgA and UapC (see Table S1). Growth is on MM supplemented with 1% glucose as C source and different nitrogen sources, such as 10 mM ammonium tartrate (NH_4_^+^), 0.5 mM uric acid (UA) or 0.5 mM hypoxanthine (HX). Thiamine presence or absence from the growth medium is depicted as +thi or –thi, respectively. (**C**) Growth tests of sphingolipid or PI biosynthesis knockdown mutants relative to isogenic controls. Details are as in (**B**). (**D**) Western blot analysis, using anti-FLAG antibodies, confirming the repression of FLAG-tagged BasA or PisA, upon thiamine addition in the growth medium. Equal loading and protein steady state levels are normalized against the amount of actin, detected with a specific antibody. (**E**) Microscopic morphology of germlings in PI, sphingolipids or ergosterol biosynthesis mutants.

For studying the role of ergosterol and its metabolic precursors (Figure 1A), we genetically disrupted the expression of the following genes: *erg11A* (AN1901), *erg11B* (AN8283), *erg5* (AN4042), *erg4A* (AN2684) and *erg4B* (AN10648), as well as, and double mutants of *erg11A/erg11B* or *erg4A/erg4B*. Erg11A and Erg11B paralogues encode putative 14-alpha sterol demethylases, Erg5 encodes a putative C-22 sterol desaturase, and Erg4 paralogues encode delta24(24-1) sterol reductases. Notice that similar to *A. fumigatus*, and dissimilar to *S. cerevisiae*, *A. nidulans* has two paralogous genes encoding the Erg11 or Erg4 enzymes. The rationale for knocking out or conditionally repressing the aforementioned genes is the following. Loss of Erg11 enzymatic activities disrupt the ergosterol pathway at a rather early biosynthetic step, leading to accumulation of metabolic sterol precursors, such as lanosterol or eburicol, which may be incorporated into membranes, but are significantly different from ergosterol in structure and physical properties (see Figure 1A). Importantly, in all fungi studied, complete knockout of Erg11 activities leads to lethality. Here, to avoid lethality in the double *erg11* mutant, one of the two *erg11* genes was conditionally repressed (*thiA_p_-erg11B*), while the second gene was totally knocked-out (*Δerg11A*). Loss of late steps in ergosterol biosynthesis (Erg5 or Erg4) have been reported to be crucial for normal fungal growth, but in most cases do not to lead to lethality. The rationale for blocking ergosterol biosynthesis at late metabolic steps was not only to avoid the severe pleiotropic effects expected in the double knockout/knock-down of Erg11, but also in order to be able to test whether ergosterol metabolic precursors that are more similar to cholesterol compared to ergosterol itself (e.g., ergosta-5,7,22/24(28)-trienol), might allow transporter biogenesis and proper function. To our knowledge, the *A. nidulans erg* genes have not been genetically modified before, but the very high identity of the relevant proteins with those of *A. fumigatus* leaves little doubt on their enzymatic activities (see Supplementary Table S3). In line with this, resistance to itraconazole (ITZ), which inhibits Erg11 activities in *A. fumigatus* and other fungi, has been shown to be conferred by extra copies of the *A. nidulans erg11* genes [54]. 

Here we recorded growth phenotypes of the mutants made on minimal media (MM) supplemented with ammonium, uric acid or hypoxanthine, as N sources. Uric acid and hypoxanthine are imported by specific, well-studied, transporters related to our interests, namely UapA and AzgA, respectively (see Introduction). Figure 1B shows that all single genetic knockout mutants of *erg* genes, as well as the double null mutant *Δerg4A/Δerg4B* were viable and formed colonies. In fact, single knockouts *Δerg4A*, *Δerg4B*, *Δerg11A* did not affect growth rate or colony formation relative to an isogenic wild-type control. Only *Δerg5*, and to a lesser degree *thiA_p_-erg11B* under repressing conditions, showed moderately reduced colony diameter and conidiation. The double knockout of *Δerg4A/Δerg4B* showed severely reduced growth, while the double *Δerg11A/thiA_p_-erg11B* mutant proved viable only when *erg11B* transcription remains derepressed but was not viable under repressing conditions (Figure 1B). As seen in the relative figure, practically the same results were obtained in all N sources tested at both 25 and 37 °C. Similar results were also obtained on several other N sources tested or different pHs. Overall, these results demonstrated that the enzymatic activities of Erg11A/B are essential for viability and that of Erg4A/B or Erg5 are also crucial for proper growth. Repression of *pisA* or *basA* by thiamine addition led to severe block in colony formation in all N sources tested (Figure 1C). Relative western blot analysis showed that the presence of thiamine for 14–16 h led to full repression of PisA and significant reduction in BasA protein steady state levels (Figure 1D). Despite incomplete repression of BasA, the reduction recorded sufficed to lead to colony growth arrest, guaranteeing that our genetic system leads to dramatic reduction in sphingolipid levels.

We tested the effect of the aforementioned lipid biosynthesis mutations on *A. nidulans* morphology at the microscopic level. Figure 1E shows that strains carrying *Δerg4A/B* or *Δerg5,* or repressible alleles of *pisA* or *basA,* were all able to germinate to germlings and young hyphae. In contrast, the double mutant *Δerg11A/thiA_p_-erg11B* produced germlings at a small degree (<10%) only under non-repressing conditions, while conidiospores did not germinate at all under repressing conditions. Noticeably, strains repressed for *basA,* or *Δerg11A/thiA_p_-erg11B* and *Δerg5* showed abnormal hyphae morphology (e.g., increased width, abnormal shape or increased branching). Rather surprisingly, the strain with repressed *pisA* or the *Δerg4A/Δerg4B* null mutant, despite causing a more severe colony growth defect compared to *Δerg5,* did not show abnormal germling morphology.

### 3.2. Subcellular Localization of UapA and AzgA Purine Transporters Is Differentially Affected in Specific Lipid Biosynthesis Mutants 

In order to investigate the effect of membrane lipids on the subcellular expression of specific transporters, namely UapA and AzgA, we constructed, via genetic crossing, strains with the aforementioned lipid biosynthesis mutations expressing fully functional versions of these transporters tagged with GFP expressed from their native promoters (see Materials and Methods). For investigating the importance of Εrg11 and Erg4 activities we constructed only the relative double mutants (*Δerg11A/thiA_p_-erg11B* and *Δerg4A/Δerg4B*), which showed a defect in growth. In all strains made, GFP tagged UapA or AzgA versions functionally replace the endogenous untagged proteins. The relative strains, besides permitting assessment of UapA or AzgA transport activity by growth tests on purines as N sources and by purine uptake assays using radiolabeled xanthine (for UapA) or adenine (for AzgA), also allow the identification of the subcellular localization of UapA and AzgA by *in vivo* epifluorescence microscopy (see below). As expected, the growth phenotypes of the lipid biosynthesis mutants expressing either UapA-GFP or AzgA-GFP were identical to the original mutants expressing the untagged versions of these purine transporters shown in Figure 1B,C. 

**Figure 2:**
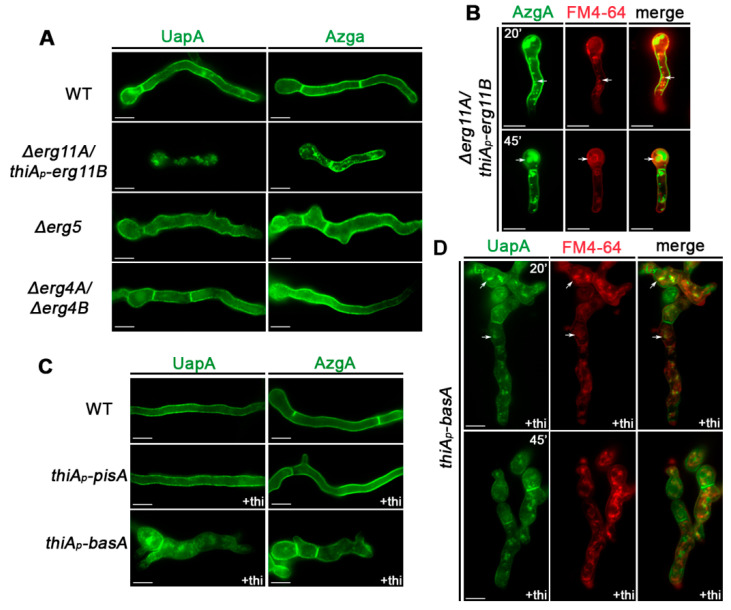
Subcellular localization of UapA and AzgA purine transporters in specific lipid biosynthesis mutants. (**A**) Epifluorescence microscopy of strains expressing UapA-GFP or AzgA-GFP from their native promoters, in different ergosterol biosynthesis mutants. Conidiospores of strains used were incubated for 16–18 h in 1% glucose MM containing the appropriate supplements and nitrate as a nitrogen source, which allow the *de novo* expression of UapA and AzgA during germination. Biological/technical replicates: 2/50 for each strain. (**B**) Co-localization of FM4-64 (red channel) with AzgA-GFP in the *Δerg11A/thiA_p_-erg11B* mutant. Notice (highlighted by arrows) that FM4-64 labels initially the PM and early endosomes appearing as cytosolic foci (20 min), and progressively vacuolar membrane rings (45 min), as previously established [46]. Biological/technical replicates: 2/30. (**C**) Epifluorescence microscopy of strains expressing UapA-GFP or AzgA-GFP from their native promoters in strains where PI or sphingolipid biosynthesis genes (*pisA* and *basA,* respectively) can be tightly repressed via *ab initio* thiamine addition (+thi). In this case, UapA and AzgA transcription was initially repressed for 14–16 h of growth through the use of ammonium as N source, and then depressed by shift in MM supplemented with nitrate as N source, for the following 4–8 h. Biological/technical replicates: 2/80 for each strain. (**D**) Co-localization of FM4-64 with UapA-GFP upon *basA* repression with thiamine. Notice the significant co-localization of FM4-64 with UapA-GFP in early endosome-like foci, highlighted with arrows. Biological/technical replicates: 2/40. The images shown represent the outcome observed in 100% of cells examined. Scale bars: 5 μm.

Figure 2 shows selected samples from a series of epifluorescence microscopic analyses of mutant and control strains, following the subcellular localization of UapA or AzgA in germlings or young hyphae. In samples lacking genes involved in the ergosterol biosynthetic pathway (*Δerg11A/thiA_p_-erg11B,* *Δerg5 and* *Δerg4A/erg4A*), UapA and AzgA expression was followed *ab initio*, that is, from the onset of germination of conidiospores (16–20 h). In cases of strains carrying repressible alleles (*thiA_p_-pisA* or *thiA_p_-basA*), UapA and AzgA transcriptional derepression was elicited by a shift from ammonium to nitrate MM media, after having established repression of relative lipid biosynthesis genes through prior addition of thiamine for 14–16 h (for details see Materials and methods and [47]). Thus, in all cases we followed the subcellular localization of newly made transporters taking place either in the absence of lipid biosynthesis genes (‘knockouts’), or after lipid biosynthesis genes have been repressed (‘knockdowns’). Notice that in a wild-type background UapA and AzgA localize cortically to PM and in septa, and do not undergo recycling or turnover via endosomes, at least under the conditions studied herein [47]. 

The knocking-out of *erg4* or *erg5* did not affect the subcellular localization or turnover of UapA of AzgA, as both transporters localize in the PM and in septal regions, similar to the wild-type control (Figure 2A). This was rather surprising given the effect of these mutations in colony growth or hyphae morphology, seen in Figure 1B,C. In the *Δerg11A/thiA_p_-erg11B* background, examined under non-repressing conditions, localization of AzgA and UapA to the PM was dramatically abolished (Figure 2A). Both UapA-GFP and AzgA-GFP label mostly cytosolic aggregates and vacuoles or foci resembling endosomes, the latter characterized by long range motility [57]. The fact that a significant fraction of AzgA still labels the PM probably reflects its higher apparent state levels compared to UapA. To further identify the nature of cytosolic structures labeled by the GFP-tagged transporters in *Δerg11A/thiA_p_-erg11B* we examined samples co-stained with FM4-64 under conditions that this lipophilic probe labels selectively endosomes and vacuolar membranes with red fluorescence. Figure 2B shows that indeed there is a significant fraction of cytosolic AzgA-GFP signal that co-localizes with FM4-64. The presence of some cytosolic structures not stained by FM4-64 suggested that these are membrane aggregates, which might arise via turnover mechanisms other than vacuolar degradation. A similar picture was obtained with UapA-GFP. Overall, these results showed that knockdown of Erg11 activities leads to aggregation and/or vacuolar turnover of both purine transporters. 

Figure 2C shows the subcellular localization of UapA-GFP or AzgA-GFP when *pisA* or *basA* transcription was repressed. Repression of PisA did not alter the capacity of these transporters to translocate and remain stable in the PM. This result is surprising given the major structural and functional role of PIs in membrane functioning in eukaryotes, but also the fact that the *pisA* repression led to arrest of growth after germling formation (see Figure 1C). Repression of BasA did not have any effect on AzgA translocation and apparent stability in the PM, despite the observed severe deformation of germling morphology and eventual arrest of growth, seen in growth tests (see Figure 1C). In contrast, reduction in BasA levels led to significant mislocalization of a fraction of UapA in cytosolic structures and membranous aggregates. Co-staining with FM4-64 showed that several of these structures are vacuoles and endosomal membranes, suggestive of dramatic turnover by endocytosis, without excluding the possibility of vacuolar sorting during trafficking (Figure 2D). 

### 3.3. Itraconazole Leads to Turnover of De Novo Made and PM-Localized UapA and AzgA Turnover, Mimicking the Effect of erg11 Knockdown

Given the severe growth arrest of the double *erg11* mutant, we used a pharmacological approach to investigate the effect of disruption of Erg11. More specifically, we employed ITZ, a standard azole antifungal, which specifically inhibits lanosterol 14α-demethylase (Erg11). We performed two kinds of epifluorescence microscopy experiments. In the first, we examined the effect of ITZ on the localization of *de novo* made UapA-GFP or AzgA-GFP. In the second, we added ITZ after UapA-GFP or AzgA-GFP was localized in the PM and further synthesis was repressed. Thus, in the first experiment we examined the effect of inhibiting Erg11 activities during transporter trafficking, while in the second we investigate whether ITZ elicits transporter internalization and turnover via endocytosis. For achieving a tight control of UapA or AzgA expression in these experiments we used strains that express these transporters through the regulatable *alcA* promoter (*alcA_p_-*UapA-GFP and *alcA_p_-*AzgA-GFP). The transcription of any gene expressed via *alcA_p_* is tightly repressed by glucose and derepressed via a shift to fructose media. Details on strain construction and conditions used to determine the effect of ITZ on UapA or AzgA localization are described in Materials and methods).

**Figure 3:**
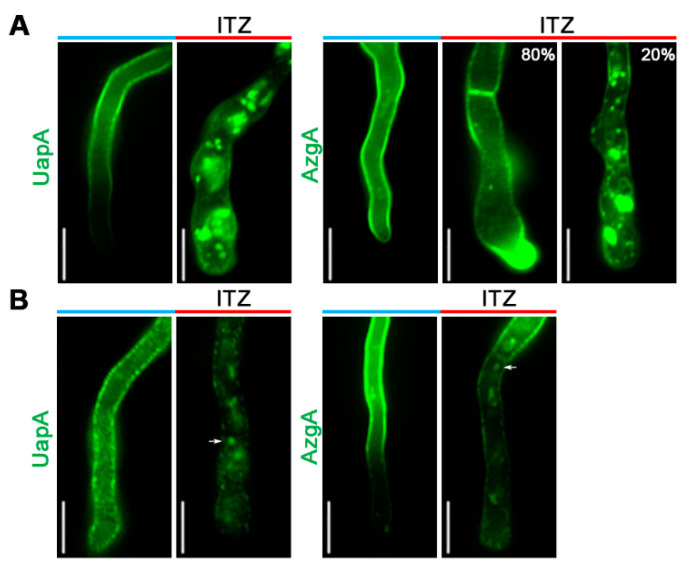
Itraconazole impact on *de novo* made and PM-localized UapA and AzgA. (**A**) Epifluorescence microscopy of strains expressing *de novo* made UapA-GFP or AzgA-GFP from the *alcA* promoter in the presence or absence of ITZ. Conidiospores germinated overnight (14 h) in 1% glucose MM to repress UapA and AzgA synthesis, and next day ITZ was added for 5 h, before shifting the culture to 0.1% fructose MM to allow derepression of UapA and AzgA for 2 h. Notice the prominent labeling of cytosolic membranous structures and foci, with concomitant reduction of PM labeling by UapA (100%), but also in a significant fraction of AzgA (20%), upon ITZ addition. Biological/technical replicates: 2/20 for each condition. (**B**) Epifluorescence microscopy showing the effect of ITZ on UapA-GFP or AzgA-GFP pre-localized in the PM. Conidiospores were initially germinated overnight (14 h) in 0.1% fructose MM to allow derepression of UapA and AzgA, and next day 1% glucose was added to the culture for 2 h to repress further synthesis of the transporters, before addition of ITZ and further incubation for 5 h. Arrows indicate highly motile foci, compatible with early endosomes and active endocytosis of the transporters. Biological/technical replicates: 2/15. Unless stated otherwise, the images shown represent the outcome observed in 100% of cells examined. Scale bars: 5 μm.

Figure 3A shows the effect of ITZ addition prior to de-repression of UapA or AzgA. UapA-GFP localization to the PM was highly affected, as florescent signals labeled mostly immotile cytosolic large aggregates, several smaller foci and membranous rings, with no evidence of PM labeling. In other words, UapA-GFP was mislocalized in endomembranes and vacuoles. A milder and more variable effect of ITZ was observed with AzgA-GFP, where only a fraction of the transported was significantly mislocalized to cytosolic structures, while in most hyphae AzgA-GFP still reached the PM. Notice that addition of ITZ under these conditions also led to modification of hyphae morphology (e.g., flattening of the tip region and increased hypha width), confirming its inhibitory effect on growth via inhibition of Erg11 activities. Figure 3B shows the effect of ITZ addition after UapA or AzgA are localized in the PM and further *de novo* synthesis was repressed. ITZ elicited internalization of both UapA and AzgA, more evident towards the younger apical regions of hyphae, especially for UapA-GFP, where PM is expected to contain increasingly reduced levels of ergosterol, given these segments developed after ITZ addition. Removal from the PM was accompanied by the appearance of small vacuolar structures and highly motile cytosolic foci, characteristic of early endosomes originated by endocytosis. Overall, results shown in Figure 3 are in agreement with the results obtained using the *Δerg11A/thiA_p_-erg11B* (Figure 2A), together establishing that Erg11 inhibition leads to defects in both sorting and stable localization of transporters in the PM. 

### 3.4. UapA and AzgA Steady State Levels Are Dramatically Reduced in erg11 Mutants, but Not When basA Is Repressed

**Figure 4:**
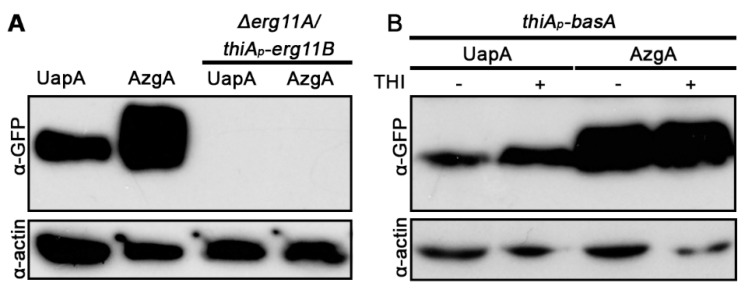
(**A**) UapA and AzgA steady state levels in *erg11* and (**B**) *basA* knockdown mutants. Western blot analysis of UapA-GFP or AzgA-GFP steady state levels using total protein extracts and monoclonal anti-GFP antibody. Actin antibody was used to calibrate protein loading. For *thiA_p_-basA*, proteins were extracted after *ab initio* (overnight) thiamine repression of BasA. Transporter expression was driven from native promoters. Notice a moderate increase in the levels UapA or AzgA levels upon *basA* repression, contrasting the full loss of UapA or AzgA detection when *erg11* activities are knocked out.

We tested the effect of selected lipid biosynthesis mutants in the protein steady state levels of UapA and AzgA. Relative western blot analysis, using anti-GFP antibody, is shown in Figure 4. No UapA or AzgA protein could be detected in the *Δerg11A/thiA_p_-erg11B* mutant, even under non-repressing conditions for *erg11B* (Figure 4A). This showed that down-regulation of an early step in ergosterol biosynthesis leads to dramatic turnover of transporters. The western blot analysis of transporters in *erg* mutants was in line with what was observed at the microscopic level, that is, *erg11* repression results in dramatic transporter turnover, trafficking and stability. Repression of *basA* did not lead to a reduction of UapA or AzgA protein levels, compared to those obtained in a *basA^+^* background (Figure 4B). This was compatible with the little effect of BasA repression in AzgA localization to the PM, but somehow contrasted the picture obtained with UapA, which showed a degree of mislocalization from the PM when basA was repressed (see Figure 2C). As will be shown later, repression of *basA* elicits turnover principally by endocytosis, and is not related to direct degradation during biogenesis. Thus, it seems that, at least under the conditions used, our western blot analysis does not detect the fraction of transporters undergoing degradation via endocytosis seen by epifluorescence analysis.

### 3.5. UapA and AzgA Transport Activities Are Not Affected by Repression of Ergosterol, Sphingolipid or PI Biosynthesis

The fact that late *erg* null mutants (*erg4A/4B, erg5*) or repression of *pisA* did not have an effect on UapA or AzgA sorting to the PM, did not exclude that, in these mutants, transporters might not function properly. In addition, in cases where UapA or AzgA trafficking is affected, but still a fraction of transporters still translocates to the PM (i.e., UapA when *basA* is repressed or AzgA in knocked out/repressed *erg11A/B*), again we could not exclude that the transporter fraction that reaches to the PM is dysfunctional, due to reduced levels of specific lipids. To address the effect of membrane lipids on transport activities per se, we performed direct uptake assays using radiolabeled substrates of UapA and AzgA (uric acid or adenine, respectively) in the background of lipid biosynthesis mutations (for details on the methodology of transport assays see Materials and methods).

Results, summarized in Table 1, lead to the following observations. First, knockdown of ergosterol, sphingolipid or PI biosynthesis did not modify significantly, at least at the physiological level, the affinity of UapA and AzgA to recognize their substrate. *K*_m_ values estimated (2–7 μΜ) remain practically unchanged compared to that obtained in the wild-type background (3–5 μΜ). Apparent transport rates (*V*) in these mutants ranged from 21–105% of the wild-type. In most cases, *V* values were moderately or not affected (68–105%). Exceptions were primarily *Δerg11A/thiA_p_-erg11B,* where UapA or AzgA transport rates were 21–38% of that of the wild-type, and when *thiA_p_-basA* was repressed (57% of the wild-type UapA rate). Notice, however, that the *V* values estimated using germlings reflect *apparent* transport rates, as they also depend on the steady state level of expression and turnover of transporters. In cases where lipid biosynthesis mutants dramatically affected cell morphology and growth (i.e., *Δerg11A/thiA_p_-erg11B* or repressed *thiA_p_-basA* and *thiA_p_*-*pisA*), any recorded reduction in transport rates may be due to transporter turnover and reduced steady state levels, rather than being due to a direct kinetic effect on transporter activity. In fact, the significant drop of apparent UapA activity in *Δerg11A/thiA_p_-erg11B* or repressed *thiA_p_-basA* was in line with the epifluorescent microscopy analysis, which confirmed a significant degree of turnover of UapA-GFP in these mutants. In agreement with epifluorescent microscopy, in all cases where UapA or AzgA localized to the PM, we detected relatively high transport activities. Notice also that in contrast to *V* values, substrate affinity constants do not depend on the amount of transporter expressed in the PM. Thus, the practically unchanged *K*_m_ values recorded in all cases, and the high transport rates recorded in mutants that did not lead to transporter degradation, point to the conclusion that the fraction of UapA or AzgA that reaches the PM is functional, even though ergosterol, sphingolipid or PI levels are apparently reduced. This rather paradoxical observation is discussed further later. 

### 3.6. Turnover of UapA in Response to Ergosterol Depletion Occurs by Multiple Mechanisms Operating during Trafficking and after PM Translocation

Wild-type UapA undergoes dramatic, activity-independent, internalization and vacuolar turnover in response to physiological (e.g., the addition of ammonium or glutamine as preferred N sources; [58]) or stress signals (addition of DMSO, DTT, antifungals or hyphal crowding; [59] and unpublished results). The primary molecular modification required for initiation of UapA endocytosis is ubiquitination at single lysine reside (Lys572), located at the cytosolic C-terminal segment of UapA. Ubiquitination is carried out by HulA, an essential Hect/Rsp5/Ned4-type ubiquitin ligase, recruited by the specific α-arrestin adaptor ArtA [48]. The genetic disruption of ubiquitination by repression or specific viable truncations of HulA, or replacement of Lys572Arg leads to UapA stabilization in the PM. Following ubiquitination, UapA is internalized by clathrin-mediated endocytosis (CME), in a process that is absolutely dependent on the key endocytic factor SagA^End3^ [46]. HulA ubiquitination is also essential for turnover of misfolded ER-retained versions of UapA via chaperone-mediated selective autophagy [60].

To dissect the mechanism by which reduction in Erg11 led to dramatic turnover of transporters, we constructed strains expressing UapA-GFP in the genetic background of mutants blocked in HECT-type ubiquitination of transporters (*hulAΔC2*), late steps of endocytosis (*ΔsagA*) or selective autophagy (*Δatg9*), and followed the effect of ITZ on UapA-GFP subcellular localization. Additionally, we also constructed and tested a strain expressing a version of UapA that cannot be ubiquitylated due to substitution of the relative Lys acceptor of ubiquitin UapA-K572R-GFP [48]. We performed experiments following the subcellular localization of newly made UapA and PM-localized, as described earlier. Strains constructed and experimental conditions used are described in Materials and methods.

**Figure 5:**
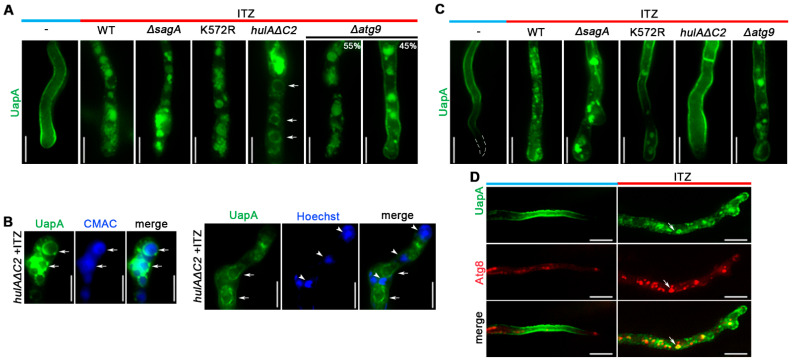
Turnover of UapA in response to ITZ in different genetic backgrounds related HulA-dependent endocytosis and selective autophagy. Details on growth conditions are identical to those described in the legend of Figure 3. In (**A**) notice that the effect of ITZ on *de novo* UapA localization, basically its mislocalization to cytosolic structures, was observed in all genetic backgrounds, although in a fraction of *Δatg9* we could still detect PM labeling by UapA. Biological/technical replicates: 2/150 for each strain. (**B**) Co-localization of UapA-GFP with CMAC (vacuoles) or Hoechst (nuclei-indicated with arrow heads) confirmed that the membranous rings (arrows) observed mostly in *hulAΔC2*, correspond to vacuolar membranes rather than perinuclear ER. Biological/technical replicates: 2/15 for each condition. (**C**) Notice the full (in *hulaΔC2*) or partial (in UapA-K572R or *Δatg9*) protection of PM pre-localized UapA from endocytic turnover. Biological/technical replicates: 2/120 for each strain. (**D**) Co-localization analysis of UapA-GFP with mCherry-Atg8 in response to ITZ addition. Notice that in the presence of ITZ, Atg8-labelled structures became more evident all over the cytoplasm and develop into hollow autophagic structures (highlighted with an arrow), and that these forms co-localize significantly with internalized UapA. Biological/technical replicates: 2/50. Unless stated otherwise, the images shown represent the outcome observed in 100% of cells examined. Scale bars: 5 μm.

Figure 5A shows that localization of *de novo* made UapA to the PM was significantly affected in all mutant backgrounds tested. However, the picture obtained in specific mutants was different. In *ΔsagA*, where endocytosis of UapA is blocked, UapA turnover by ITZ was severe, compatible with labeling of large vacuoles and other membrane aggregates. A similar, but not identical, picture compatible with mislocalization from the PM was obtained with UapA-K572R-GFP or in the *hulAΔC2* background, where we also detected that UapA-GFP labels prominent membranous rings, which proved to be vacuolar membranes, as they encircle CMAC-staining structures, unrelated to perinuclear ER (see Figure 5B). Thus, it seems that when HulA ubiquitination of UapA is blocked, the transporter is still sorted towards vacuoles, but cannot translocate efficiently into their lumen for degradation. In *Δatg9,* a fraction of UapA (~45%) is localized in the PM, suggesting that selective autophagy might operate during biogenesis of UapA and thus contributes to turnover. 

When we examine the effect of ITZ on UapA pre-localized in the PM we noticed that in *ΔsagA* a significant fraction of UapA remained localized in the PM, but nevertheless a high degree of vacuolar degradation was detected (see Figure 5). This suggested that there has been internalization of UapA by a SagA-independent mechanism. Notably, this internalization was reduced in UapA-K527R-GFP and totally abolished in *HulAΔC2*, where ‘canonical’ UapA ubiquitination is in principle blocked. Thus, HulA-dependent ubiquitination of UapA pre-localized in the PM seems to be the principal modification triggered by ITZ for initiating transporter internalization via both SagA-independent and SagA-dependent mechanisms. Interestingly, the observation that genetic inactivation of *atg9* significantly reduced UapA endocytosis in all germlings strongly suggest that selective autophagy, operating at the level of the PM, might account for the SagA-independent mechanism of internalization, when ergosterol levels are reduced. To address this issue in more detail, we performed co-localization experiments using a marker of autophagosomes, namely mCherry-Atg8 [57], expressed in parallel with UapA-GFP. In the absence of ITZ, we detected a low degree of autophagy (i.e., low amounts of mCherry-Atg8), which dramatically increased in the presence of ITZ (Figure 5D, middle panel). Notably, UapA-GFP and mCherry-Atg8 co-localized in several cytosolic structures resembling mature autophagosomes (lower panel in Figure 5D). This confirmed the involvement of selective autophagy in the turnover of internalized from the PM UapA.

### 3.7. BasA-Dependent Turnover of UapA Occurs by HulA-Dependent Endocytosis and Selective Autophagy

**Figure 6:**
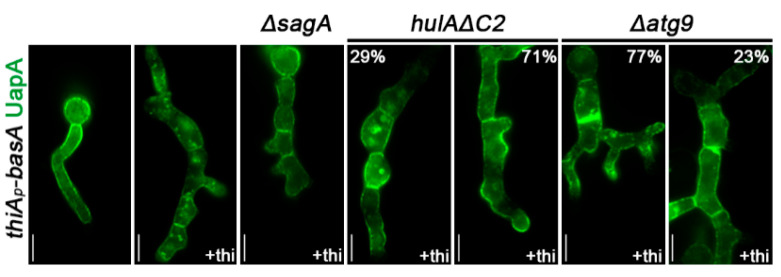
BasA-dependent localization of UapA in mutants blocked HECT-type endocytosis, ubiquitination or selective autophagy. The figure shows epifluorescence microscopy images of strains expressing *de novo* made UapA-GFP in *ΔsagA, hulaΔC2* and *Δatg9* genetic backgrounds. Details of conditions used are as those described in the legend of Figure 2C. Notice the full or partial protection of UapA from endocytosis in *ΔsagA* and *hulaΔC2* or *Δatg9,* respectively. Biological/technical replicates 2/120 for each strain. Unless stated otherwise, the images shown represent the outcome observed in 100% of cells examined. Scale bars: 5 μm.

We also tested the role of endocytosis, HulA ubiquitination and selected autophagy on UapA turnover upon BasA knockdown after *ab initio* establishment of transcriptional repression of the *basA* gene (Figure 6). Blocking endocytosis via deletion of *sagA* fully rescued sorting and stable localization to the PM of *de novo* made UapA-GFP. Lack of HulA-dependent ubiquitination (hulAΔC2) also rescued *de novo* made UapA-GFP translocation and stability in the PM, in the majority of samples (~71%). Thus, UapA turnover in response to reduction in sphingolipid levels was principally due to endocytosis. Relating the picture obtained in *ΔsagA* and *hulAΔC2* we noticed that a fraction of UapA undergoes internalization in the absence of HulA activity, probably by exposing the transporter to a distinct internalization mechanism, probably selective autophagy, as in *Δatg9* ergosterol depletion reduced the degree of UapA mislocalization (23%) and turnover to a degree that is close to HulA-independent internalization (29%).

## 4. Conclusions

Our present work investigated, for the first time, the effect of genetic blocks or reduction of enzyme activities involved in the biosynthesis of specific membrane lipids on *A. nidulans* growth and specifically on the biogenesis, activity and turnover of selected solute transporters, namely UapA and AzgA. Both these nutrient carriers have been extensively studied in our laboratory and particularly UapA is one the best characterized eukaryotic transporters at all possible levels [35]. Our interest on the role of membrane lipids in transporter function stemmed also from two recent findings. First, PI lipids seem to be critical for ER exit and functional dimer formation of UapA [11,38]. Second, UapA, AzgA and other transporters of *A. nidulans* are sorted from the ER to the PM via Golgi-bypass through a rather unexpected mechanism, where the role of lipids remains elusive [47]. 

The present work led to a series of novel findings. Firstly, and rather surprisingly, transcriptional repression of PI synthase (PisA) led to colony growth arrest, but did not affect transporter subcellular localization and stability in germlings, prior to the eventual growth arrest (i.e., for at least 20 h following conidiospore germination and 10 h after tight repression of *pisA*). Interestingly, PisA repression also did not affect the trafficking of a number of apical markers, such as chitin synthase and syntaxin A (unpublished observations). This is quite surprising given the importance of PIs in cell growth in all organisms studied. Even more unexpectedly PisA repression did not affect UapA or AzgA transport kinetics. In [38] it has been shown that addition of PIs restores UapA dimerization in purified delipidated UapA monomers, supporting the notion that PIs are essential for transport activity. The present work, however, suggests that reduction in PI levels during germination is not detrimental to transporter subcellular trafficking, activity and stability. In addition to the redundancy of PisA activity in transporter function, mutations affecting ergosterol or sphingolipids also did not affect UapA or AzgA transport kinetics. This is best reflected by the observation that *K*_m_ values of UapA and AzgA measured in all mutant backgrounds are very similar to those measured in isogenic wild-types control strains. It is thus very probable that *in vivo* other phospholipids/lipids compensate for the reduction in specific lipids [14]. For example, lipid compensation has been previously reported in a specific case where a dominant mutation in acyltransferase Slc1 permits this enzyme to synthesize unusual lipids containing a C26 fatty acid attached to PIs. These C26-containing PIs mimic sphingolipids and can restore the PM expression of Pma1 [31]. 

A second important finding is that early block in ergosterol biosynthesis, specifically repression or inactivation of Erg11 by ITZ, proved detrimental to transporter trafficking, localization and stability in the PM. In contrast, genetic inactivation of the *erg4* and *erg5* genes, involved in the last two steps of ergosterol biosynthesis, had practically no effect on transporter biogenesis and function, despite leading to altered growth rate and morphology of the relevant mutant colonies. This means that transporter function does not necessitate ergosterol per se, suggesting that metabolic precursors of ergosterol can compensate for transporter function. This point might be exploited in future efforts to heterologously express in *A. nidulans* transporters from metazoa and other organisms containing sterols more similar to specific ergosterol precursors rather than ergosterol itself.

A third interesting observation is that while an early ergosterol defect affects pleiotropically transporter biogenesis and turnover, repression of sphingolipid biosynthesis leads mainly to transporter turnover by internalization from the PM. Similarly, sphingolipid genetic depletion has been shown previously to trigger the endocytic turnover of Gap1 [27]. Given that our transcriptional knockdown system concerning BasA expression does not fully deplete *A. nidulans* germlings from sphingolipids, we cannot exclude that sphingolipids may also have a role in sorting of *de novo* made transporters to the PM, as reported in the case of Tat2 or Fur4 in yeast [27]. An interesting finding was that sphingolipid depletion-elicited endocytosis of UapA proved partly independent of HulA-associated ubiquitination, suggesting the parallel involvement of alternative mechanisms of internalization and turnover, such as selective autophagy, operating at the level of the PM [61,62,63]. Notably also, and differently from Gap1, the UapA fraction that reaches the PM under sphingolipid depletion shows significant apparent transporter activity and normal substrate affinity. 

A last notable point of this work is the somehow differential sensitivity of the two transporters studied to membrane lipid biosynthesis defects. AzgA was clearly more resistant than UapA. UapA is formally shown to form tight dimers *in vivo* [36]. In contrast, we have failed to obtain experimental evidence for AzgA dimerization. Molecular modeling of AzgA also shows that AzgA is distinctively different from UapA, as far as it concerns specific domains, as for example TMS13 and TMS14, which in UapA are involved in dimerization (Emmanuel Mikros and George Diallinas, unpublished observations). Finally, UapA and AzgA also show different degrees of sensitivity to transport-dependent or ammonium-elicited endocytosis, UapA being the most sensitive [48], as also shown here in response to membrane lipid defects. A similar observation has been made in yeast, where Fur4 and Pma1 are not equally sensitive to changes in the lipid environment, which in this case has been attributed also to differences in their oligomerization status [28,29,30,31,33]. 

## Figures and Tables

**Table 1:** Transport kinetics of UapA and AzgA in lipid biosynthesis mutants. Results shown represent averages of two independent experiments, using measurement from triplicates samples. UapA and AzgA subcellular localization, determined by epifluorescence microscopy in strains expressing GFP-tagged UapA or AzgA grown under the same conditions of transport assays, are also shown.

	Xanthine Uptake Kinetics		UapA Localization	Adenine Uptake Kinetics		AzgA Localization
Allele	*K*_m_ (μΜ)	*V* (%)		*K*_m_ (μΜ)	*V* (%)	
wild-type	5 ± 1	100 ± 12	PM	3 ± 1	100 ± 9	PM
*Δerg4A/Β*	4 ± 1	68 ± 14	PM	n.d.	n.d.	PM
*Δerg5*	4 ± 2	101 ± 12	PM	n.d.	n.d.	PM
*Δerg11A*	n.d.	105 ± 10	n.d.	n.d.	n.d.	n.d.
*thiA_p_-erg11B* *	n.d.	95 ± 5	n.d.	n.d.	n.d.	n.d.
*Δerg11A/* *thiA_p_-erg11B* *	2 ± 1	21 ± 3	cytoplasmic puncta/foci	7 ± 2	38 ± 9	PM/cytoplasmic puncta/ foci
*thiA_p_-basA* *	7 ± 1	57 ± 6	PM/cytoplasmic puncta/ foci	2 ± 1	99 ± 10	PM
*thiA_p_-pisA* *	2 ± 1	94 ± 14	PM	2 ± 1	104 ± 12	PM

* thiamine addition for 6 h; n.d.—not determined; PM—Plasma Membrane.

## Data Availability

Not applicable.

## Supplementary Materials

The following are available online at https://www.mdpi.com/article/10.3390/jof7070514/s1, Table S1: *A. nidulans* strains used and constructed in this study. Table S2: Oligonucleotides used in this study for cloning or gene disruptions. Table S3: Identity (similarity) of *A. nidulans* enzymes involved in membrane lipid biosynthesis relevant to the present work with orthologues form *A. fumigatus* and *S. cerevisiae*.
