# Supplementary material for: Impact of Membrane Lipids on UapA and AzgA Transporter Subcellular Localization and Activity in Aspergillus nidulans
Source: J Fungi (Basel). 2021 Jun 28;7(7):514. doi: 10.3390/jof7070514 (PMC8304608; doi:10.3390/jof7070514)
Supplement: Supplementary file 1 [file jof-07-00514-s001.zip › Supplementary Table S1.pdf]

Supplementary Table S1: *Aspergillus nidulans* strains used and constructed during this study. All strains also carry the veA1 mutation affecting sporulation. pabaA1, pyroA4, riboB2, argB2 and pyrG89/pyr4 are auxotrophic mutations for p-aminobenzoic acid, pyridoxine, riboflavin, arginine and uracil/uridine, respectively.

**Strain**	**Genotype**	**Reference**
TNO2A7	*nkuAΔ::argB pyrG89 pyroA4 riboB2*	[1]
wt	*pabaA1*	FGSC*
Δ3	*uapAΔ uapCΔ::AFpyrG azgAΔ argB2 pabaA1*	[2]
azgA-GFP	*(pGEM)azgA-GFP uapAΔ uapCΔ::AFpyrG azgAΔ pabaA1*	[2]
alcAp-azgA-GFP	*(pGEM-alcAp-panB)alcAp-azgA-GFP uapAΔ uapCΔ::AFpyrG azgAΔ fcyBΔ::argB furDΔ::AFriboB furAΔ::AFriboB cntAΔ::AFriboB pantoB100 pabaA1*	[3]
uapA-GFP	*uapAΔ::uapA-GFP::AFriboB uapCΔ::AFpyrG nkuAΔ::argB pabaA1 pyroA4 riboB2*	[4]
alcAp-uapA-GFP	*uapAΔ::alcAp::uapA-GFP::AFriboB uapCΔ::AFpyrG nkuAΔ::argB pabaA1 pyroA4 riboB2*	[4]
Δerg11A	*AN1901Δ::AFpyrG nkuAΔ::argB pyrG89 pyroA4 riboB2*	this study
thiAp-erg11B	*thiAp-AN8283::AFpyrG nkuAΔ::argB pyrG89 pyroA4 riboB2*	this study
Δerg11A/thiAp-erg11B	*AN1901Δ::AFpyrG thiAp-AN8283::AFriboB nkuAΔ::argB pyrG89 pyroA4 riboB2*	this study
Δerg4A	*AN2684Δ::AFpyrG nkuAΔ::argB pyrG89 pyroA4 riboB2*	this study
Δerg4B	*AN10648Δ::AFpyrG nkuAΔ::argB pyrG89 pyroA4 riboB2*	this study
Δerg4A/Δerg4B	*AN2684Δ::AFpyrG AN10648Δ::AFriboB nkuAΔ::argB pyrG89 pyroA4 riboB2*	this study
Δerg5	*AN4042Δ::AFpyrG nkuAΔ::argB pyrG89 pyroA4 riboB2*	this study
Δerg11A/thiAp-erg11B uapA-GFP	*AN1901Δ::AFpyrG thiAp-AN8283::AFriboB uapAΔ::uapA-GFP nkuAΔ::argB pabaA1*	this study
Δerg11A/thiAp-erg11B azgA-GFP	*AN1901Δ::AFpyrG thiAp-AN8283::AFriboB uapAΔ azgA-GFP pabaA1 pyroA4*	this study
Δerg4A/Δerg4B uapA-GFP	*AN2684Δ::AFpyrG AN10648Δ::AFriboB nkuAΔ::argB uapA-GFP pyrG89 pabaA1*	this study
Δerg4A/Δerg4B azgA-GFP	*AN2684Δ::AFpyrG AN10648Δ::AFriboB azgA-GFP nkuAΔ::argB pyrG89 pyroA4 riboB2*	this study
Δerg5 uapA-GFP	*AN4042Δ::AFpyrG uapAΔ::uapA-GFP nkuAΔ::argB pyrG89 pyroA4*	this study
Δerg5 azgA-GFP	*AN4042Δ::AFpyrG nkuAΔ::argB azgA-GFP pyrG89 pyroA4 pabaA1*	this study
thiAp-basA	*thiAp-AN0640::AFpyrG nkuAΔ::argB pyrG89 pyroA4 riboB2*	[5]
thiAp-FLAG-basA	*thiAp-FLAG-AN0640::AFpyrG nkuAΔ::argB pyrG89 pyroA4 riboB2*	[5]
thiAp-basA uapA-GFP	*thiAp-AN0640::AFpyrG uapAΔ::uapA-GFP nkuAΔ::argB pyrG89 pyroA4 riboB2*	[5]
thiAp-basA azgA-GFP	*thiAp-AN0640::AFpyrG azgA-GFP pabaA1*	[5]
artAΔ	*AN0056Δ::AFriboB nkuAΔ::argB pyrG89 pyroA4 riboB2*	[6]
sagAΔ	*AN1023Δ::AFriboB nkuAΔ::argB pyrG89 pyroA4 riboB2*	[6]
hulAΔC2	*uapAΔ uapCΔ::AFpyrG azgAΔ HulA::pyr4-hulAΔC2 pabaA1*	[7]
thiAp-basA uapA-GFP artAΔ	*thiAp-AN0640::AFpyrG uapAΔ::uapA-GFP artAΔ::AFriboB nkuAΔ::argB pabaA1*	this study
thiAp-basA uapA-GFP SagAΔ	*thiAp-AN0640::AFpyrG nkuAΔ::argB uapAΔ::uapA-GFP sagAΔ::AFriboB pabaA1*	this study
thiAp-basA uapA-GFP hulAΔC2	*thiAp-basA::AFpyrG uapAΔ::uapA-GFP nkuAΔ::argB uapCΔ::AFpyrG hulA::pyr4-hulAΔC2 pabaA1*	this study
thiAp-pisA	*thiAp-AN0913::AFpyrG nkuAΔ::argB pyrG89 pyroA4 riboB2*	this study
thiAp-FLAG-pisA	*thiAp-FLAG-AN0913::AFpyrG nkuAΔ::argB pyrG89 pyroA4 riboB2*	this study

thiAp-pisA uapA-GFP	*thiAp-AN0913::AFpyrG uapAΔ::uapA-GFP nkuAΔ::argB pyrG89 pyroA4*	this study
thiAp-pisA azgA-GFP	*thiAp-AN0913::AFpyrG azgA-GFP pabaA1*	this study
thiAp-pisA alcAp-azgA-GFP	*thiAp-AN0913::AFpyrG alcAp-azgA-GFP pabaA1*	this study
alcAp-uapA-GFP	*(pBS-alcAp-argB)alcAp-uapA-GFP uapAΔ uapCΔ::AFpyrG azgAΔ pabaA1 argB2*	[7]
alcAp-uapA-GFP sagAΔ	*(pBS-alcAp-argB)alcAp-uapA-GFP nkuAΔ::argB sagAΔ::AFriboB pabaA1*	[6]
alcAp-uapA-Δ543-574-GFP	*(pBS-alcAp-argB)alcAp-uapA-Δ543-574-GFP uapAΔ uapCΔ::AFpyrG azgAΔ pabaA1 argB2*	[7]
alcAp-uapA-K572R-GFP	*(pBS-alcAp-argB)alcAp-uapA-K572R-GFP uapAΔ uapCΔ::AFpyrG azgAΔ pabaA1 argB2*	[7]
alcAp-uapA-GFP hulAΔC2	*(pBS-alcAp-argB)alcAp-uapA-GFP uapAΔ uapCΔ::AFpyrG azgAΔ pabaA1 hulA::pyr4-hulAΔC2*	[7]
alcAp-uapA-GFP Δatg9	*(pBS-alcAp-argB)alcAp-uapA-GFP atg9Δ::AFpyrG nkuAΔ::argB pabaA1 pyroA4*	this study
alcAp-UapA-GFP mCherry-atg8	*pyroA4[pyroAΔ::gpdAm-mCherry-atg8] uapAΔ::alcAp-UapA-GFPL::AFriboB pabaA1*	[4]

*FSCG: http://www.fgsc.net

1. Nayak, T.; Szewczyk, E.; Oakley, C.E.; Osmani, A.; Ukil, L.; Murray, S.L.; Hynes, M.J.; Osmani, S.A.; Oakley, B.R. A Versatile and Efficient Gene-Targeting System for Aspergillus nidulans. *Genetics* **2006**, *172*, 1557–1566, doi:10.1534/genetics.105.052563.

2. Pantazopoulou, A.; Lemuh, N.D.; Hatzinikolaou, D.G.; Drevet, C.; Cecchetto, G.; Scazzocchio, C.; Diallinas, G. Differential physiological and developmental expression of the UapA and AzgA purine transporters in Aspergillus nidulans. *Fungal Genet. Biol.* **2007**, *44*, 627–640, doi:10.1016/j.fgb.2006.10.003.

3. Dimou, S.; Diallinas, G. Life and Death of Fungal Transporters under the Challenge of Polarity. *Int. J. Mol. Sci.* **2020**, *21*, doi:10.3390/ijms21155376.

4. Evangelinos, M.; Martzoukou, O.; Chorozian, K.; Amillis, S.; Diallinas, G. BsdA Bsd2 -dependent vacuolar turnover of a misfolded version of the UapA transporter along the secretory pathway: prominent role of selective autophagy. *Mol. Microbiol.* **2016**, *100*, 893–911, doi:10.1111/mmi.13358.

5. Martzoukou, O.; Amillis, S.; Zervakou, A.; Christoforidis, S.; Diallinas, G. The AP-2 complex has a specialized clathrin-independent role in apical endocytosis and polar growth in fungi. *Elife* **2017**, *6*, doi:10.7554/eLife.20083.

6. Karachaliou, M.; Amillis, S.; Evangelinos, M.; Kokotos, A.C.; Yalelis, V.; Diallinas, G. The arrestin-like protein ArtA is essential for ubiquitination and endocytosis of the UapA transporter in response to both broad-range and specific signals. *Mol. Microbiol.* **2013**, *88*, 301–317, doi:10.1111/mmi.12184.

7. Gournas, C.; Amillis, S.; Vlanti, A.; Diallinas, G. Transport-dependent endocytosis and turnover of a uric acid-xanthine permease. *Mol. Microbiol.* **2010**, *75*, 246–260, doi:10.1111/j.1365-2958.2009.06997.x.
