# Supplementary material for: Impact of Membrane Lipids on UapA and AzgA Transporter Subcellular Localization and Activity in Aspergillus nidulans
Source: J Fungi (Basel). 2021 Jun 28;7(7):514. doi: 10.3390/jof7070514 (PMC8304608; doi:10.3390/jof7070514)
Supplement: Supplementary file 1 [file jof-07-00514-s001.zip › Supplementary Table S2.pdf]

Supplementary Table S2: Oligonucleotides used in this study for cloning and gene disruption purposes.

Primer	Sequence 5′-3′
AN1901 5F	CGTTTCTTCGCTGCCACTTGCACC
AN1901 5R	GGAGGTTGTTCAGACCATCGATG
AN1901 3F	GATTTCAAGATCCCGTTAAGCCTG
AN1901 3R	CCAGCATGTTATGCGGGCTACAAG
AN1901 5R pyrG	CATCGATGGTCTGAACAACCTCCGCCTCAAACAATGCTCTTCACCC
AN1901 3F pyrG	CAGGCTTAACGGGATCTTGAAATCCTGTCTGAGAGGAGGCACTGATG
AN1901 5F nested	CTTCGCGAAGTTATGGATATTGCTCG
AN1901 3R nested	CTCCAGATTCAAGTCGGCTGCAG
AN8283 5 ApaI F	CGCGGGGCCCGAGGTTACTACGAGTCTCTGATGAAC
AN8283 5 SpeI R	CGCGACTAGTCGTATCCATTTGTATGTCCGTTGCGG
AN8283 ORF SpeI F	CGCGACTAGTATGGGACTTGTCTCCCTTGTCCTCG
AN8283 ORF NotI R	CGCGGCGGCCGCCTGTTGGTACCCTTGCTGACCAGAC
AN2684 5 ApaI F	CGCGGGGCCCCCAACCATTCAGGATCGGATCGG
AN2684 5 XbaI R	CGCGTCTAGAGCTGCGCCATTGCCTCTGATGTCAGC
AN2684 3 XbaI F	CGCGTCTAGAGGTGGATCGATCTGCACCTATACC
AN2684 3 NotI R	CGCGGCGGCCGCCACATGCGAAATGTCTGGACCAG
AN10648 5 ApaI F	CGCGGGGCCCGTGAACCGGCCACCAAAGGATCC
AN10648 5 SpeI R	CGCGACTAGTGATTGCGAAAACATGCGAGAATTGAC
AN10648 3 SpeI F	CGCGACTAGTCCAGTACGTGTTTTAGGAGTGTTGC
AN10648 3 NotI R	CGCGGCGGCCGCCTCTACCTAGATACCTAGGCTAGG
AN4042 5 ApaI F	CGCGGGGCCCGTATGGATCTCAAACATGCCTTCGC
AN4042 5 SpeI R	CGCGACTAGTCTTGAGCGATTGAACGGACCCCAG
AN4042 3 SpeI F	CGCGACTAGTCTTGTACCGCAATGCAACAGCAAC

AN4042 3 NotI R	CGCGGCGGCCGCCTTCACAACTTGCTTGCGCTGACG
AN0913 5 ApaI F	CGCGGGGCCCGGACTTTGTCTCCGCCCAAGC
AN0913 5 XbaI R	CGCGTCTAGACAGTTACTGTCAGCTTGGAGGGC
AN0913 ORF XbaI F	CGCGTCTAGAATGAGCGCTCGTACCAGAAGGCAG
AN0913 3 NotI R	CGCGGCGGCCGCGGGCGGTTATGTACGGTAATCCG
AFpyrG SpeI F	CGCGACTAGTGCCTCAAACAATGCTCTTCACCC
AFpyrG SpeI R	CGCGACTAGTCTGTCTGAGAGGAGGCACTGATG
AFpyrG XbaI F	CGCGTCTAGAGCCTCAAACAATGCTCTTCACCC
AFpyrG XbaI R	CGCGTCTAGACTGTCTGAGAGGAGGCACTGATG
AFriboB SpeI F	CGCGACTAGTAAGCTTGATATCACAATCAGCTTTTC
AFriboB SpeI R	CGCGACTAGTCCCGGGCTGCAGGAATTCGATAAG
thiAp SpeI F	CGCGACTAGTCGACCTGGCACCTACAGAAGAATCC
thiAp SpeI R	CGCGACTAGTGTTGACTCAGTTCAATGGTTCGAC
thiAp FLAG SpeI R	CGCGACTAGTCTTGTCATCGTCGTCCTTGTAGTCCATGTTGACTCAGTTCAATGGTTC
