# Supplementary material for: Impact of Membrane Lipids on UapA and AzgA Transporter Subcellular Localization and Activity in Aspergillus nidulans
Source: J Fungi (Basel). 2021 Jun 28;7(7):514. doi: 10.3390/jof7070514 (PMC8304608; doi:10.3390/jof7070514)
Supplement: Supplementary file 1 [file jof-07-00514-s001.zip › Supplementary Table S3.pdf]

Supplementary Table S3. For gene identities and annotation see https://fungidb.org/fungidb/app

Aspegillus nidulans	*Aspergillus fumigatus*					*Saccharomyces cerevisiae*
	Erg11A Afu5g14350	Erg11B Afu1g07140	Erg4A Afu4g03630	Erg4B Afu4g06890	Erg5 Afu7g03740	Pis1 YPR11W
Erg11A AN1901	73.6% **(90.6%)**	60.8% (82.9%)				
Erg11B AN8283	62.0% (83.3%)	79.7% **(94.1%)**				
Erg5 AN4042					79.3% **(93.1%)**	
Erg4A AN2684			70.7% **(87.0%)**	67.9% (86.5%)		
Erg4B AN10648			68.0% (86.5%)	80.7% **(92.9%)**		
PisA AN0913						52.0% **(74.6%)**
